# Eradication of *Helicobacter pylori* may improve dyspepsia in the elderly for the long term

**DOI:** 10.1186/s12876-021-02027-6

**Published:** 2021-11-25

**Authors:** Ikko Tanaka, Shoko Ono, Yoshihiko Shimoda, Masaki Inoue, Sayoko Kinowaki, Momoko Tsuda, Masayoshi Ono, Keiko Yamamoto, Yuichi Shimizu, Mototsugu Kato, Naoya Sakamoto

**Affiliations:** 1 Department of Gastroenterology, Hokkaido University Graduate School of Medicine, Sapporo, Hokkaido Japan; 2 Department of Gastroenterology, Hokkaido University Hospital, Sapporo, Hokkaido Japan; 3 Department of Gastroenterology, National Hospital Organization Hakodate National Hospital, Hakodate, Hokkaido Japan; 4 Division of Endoscopy, Hokkaido University Hospital, Sapporo, Hokkaido Japan

**Keywords:** Dyspepsia, *Helicobacter pylori*, Eradication therapy, The elderly

## Abstract

**Background:**

Therapy for eradication of *Helicobacter pylori* (*H. pylori*) improves symptoms of *H. pylori*-associated dyspepsia (HPD), but the effects of eradication in elderly patients are unclear. The aim of our study was to investigate dyspepsia symptoms and long-term effects of eradication in elderly patients.

**Methods:**

This retrospective study included 496 patients who received *H. pylori* eradication therapy. The patients were divided into a group of elderly patients (group E: ≧ 65 years old) and a group of non-elderly patients (group N: < 65 years old). Abdominal symptoms were evaluated using a questionnaire about abdominal symptoms before eradication and after eradication (1–2 months and more than one year). Dyspepsia was defined as a score of 4 points or more for at least one of 4 items (postprandial fullness, early satiety, epigastric pain, and hunger pain). Improvement of symptoms was defined on the basis of changes in Global Overall Systems scores.

**Results:**

There were no differences in abdominal symptoms before eradication between the two groups. Successful eradication improved symptoms in patients with dyspepsia within 2 months (in 75.6% (56/74) of the patients in group N and in 64.5% (20/31) of the patients in group E). The questionnaire showed that 80% (32/40) of the patients in group N and 60% (12/20) of the patients in group E had long-term relief of dyspepsia. The scores for abdominal symptoms in group E continued to improve for a mean period of 54.8 months after eradication.

**Conclusions:**

Eradication of *H. pylori* age-independently improved dyspepsia symptoms for the long term.

**Supplementary Information:**

The online version contains supplementary material available at 10.1186/s12876-021-02027-6.

## Background

Functional dyspepsia (FD) is defined in the ROMA IV criteria as one or more of the following symptoms persisting for the past 3 months with symptom onset at least 6 months ago: postprandial fullness, early satiation, epigastric pain and epigastric burning [1]. *Helicobacter pylori* (*H. pylori*) infection is often associated with dyspepsia symptoms, and it has been reported that eradication of *H. pylori* improved the symptoms [2–4]. In the Kyoto Global Consensus Report in 2015, it was stated that all *H. pylori*-positive individuals worldwide should receive eradication therapy [5]. Furthermore, *H. pylori*-associated dyspepsia (HPD) is defined in that report as sustained symptomatic relief for 6 to 12 months after eradication, because the symptomatic gain takes at least 6 months to become significant over no eradication and this has been attributed to the time it takes for gastritis to recover [5].

In Japan, *H. pylori* infection is one of the major infections, especially in elderly people [6]. Mamori et al. reported that the rate of successful eradication of *H. pylori* in first-line therapy was lower in patients less than 50 years of age than in patients aged over 50 years [7]. However, Kobayashi et al. reported that age did not affect the efficacy or safety of eradication therapy [8]. Pilotto et al. also reported that outcomes of eradication and incidence of adverse events for the elderly were the same as those on non-elderly people [9, 10]. However, there have been few reports on the effect of *H. pylori* eradication on dyspepsia symptoms in elderly people. In this study, we evaluated the outcomes of *H. pylori* eradication therapy and followed the long-term effect on dyspepsia symptoms in elderly patients.

## Methods

### Patients

Consecutive patients who visited our *H. pylori*-specific out-patient unit and received eradication therapy during the period from January 2009 to December 2017 were retrospectively analyzed. Esophagogastroduodenoscopy revealed no active gastric diseases before eradication in any of the patients. We divided the patients into two groups according to age: an elderly group (group E) of patients who were 65 years of age or older and a non-elderly group (group N) of the patients who were less than 65 years of age. The study was approved by the Ethics Committee of Hokkaido University Hospital (Approval Number 018-0367). All patients provided written informed consent.

### *H. pylori* test

Before eradication, both a ^13^C-urea breath test (UBT) (Ubit®, Otsuka Pharmaceutical, Tokyo, Japan) and one or more other *H. pylori* tests (rapid urease test, serological and urinary anti-*H. pylori* IgG antibody, culture and microscopic examination) were used. Generally, patients in whom one of the tests was positive were defined as positive for *H. pylori*.

Successful eradication was confirmed using the UBT at 1 to 2 months after the completion of eradication treatment. When the values of UBT were negative before eradication and when the values of UBT were weakly positive (2.5 to 5.0‰, cut-off value: 2.5‰) after eradication, we confirmed the results of other tests.

### Eradication regimen

The prescribed regimens during the study period are summarized in Additional file 1: Table S1. Vonoprazan (VPZ) has been available since March 2015 in our institution and proton-pump inhibitors (PPIs) were changed to VPZ after it became available.

### Evaluation of upper gastrointestinal symptoms

A questionnaire with a scale from 1 (no problem) to 7 (very severe problem) consisting of 17 items covering Global Overall Systems (GOS) and Gastrointestinal Symptom Rating Scale was used [11, 12]. The questionnaire was filled out by each patient before the UBT. Patients who had a score of 4 points or more for at least one of 4 items (postprandial fullness, early satiety, epigastric pain, and hunger pain) were defined as patients with dyspepsia before eradication (1st questionnaire). Improvement of dyspepsia was defined as a decrease in the maximum score of abdominal symptoms before eradication by more than 2 points and each GOS item after eradication therapy being less than 3 points. For evaluation of upper gastrointestinal (GI) symptoms, that GOS questionnaires are simple and valid outcome measurements to assess the symptoms of FD according to the severity of the following eight symptoms: epigastric pain, heartburn, acid reflux, stomach discomfort, nausea, belching, early satiety and distention [10, 13, 14]. The 2nd questionnaire was given to patients on the day of judgement.

For evaluation of the long-term effects *H. pylori* eradication on dyspepsia symptoms, the 3rd questionnaire was given to patients with defined dyspepsia before eradication for whom more than 1 year had passed after successful eradication. HPD was defined as sustained dyspepsia relief for more than 1 year after successful eradication and it was confirmed by the 3rd questionnaire.

### Measured outcome parameters

The primary endpoint was long-term improvement in the GOS score after successful *H. pylori* eradication in elderly patients with dyspepsia. Secondary endpoints were successful eradication rates, adverse events, and short-term and long-term improvements of each GOS item in groups E and N.

Analysis of *H. pylori* eradication efficacy was performed on an intention-to-treat (ITT) basis. Compliance with therapy and adverse events were determined by a questionnaire at the time of judgement of *H. pylori* eradication.

### Sample size

Based on an expected 25% difference in long-term improvement of dyspepsia between young patients (50%) and elderly patients (25%) using G power (α = 0.05, β = 0.2) from previous our data, we estimated that a sample size of 132 patients (66 patients in each group) would be sufficient to demonstrate a significant difference.

### Statistical analysis

Mean values were calculated for continuous variables and percentages were calculated for categorical data. Categorical data were compared using Fisher's exact test and numerical data were compared using Student’s *t*-test. A *P* value of < 0.05 in each analysis was considered statistically significant.

## Results

### Outcomes of *H. pylori* eradication therapy

**Fig. 1:**
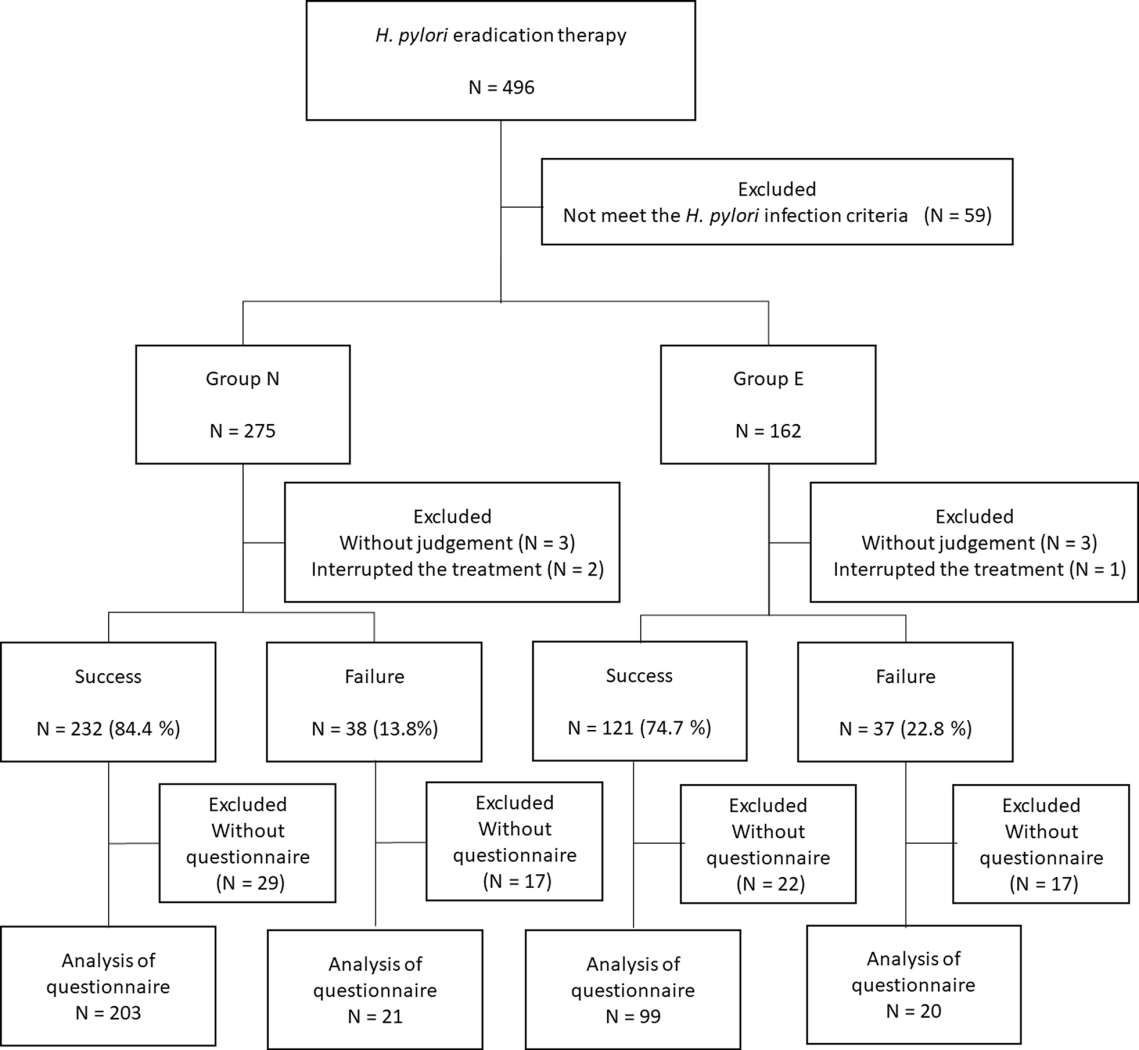
Flow diagram of patients

A total of 496 patients received *H. pylori* eradication therapy during the study period. Fifty-nine patients who did not meet our criteria for diagnosis of *H. pylori* infection were excluded, and finally a total of 437 patients including 275 patients in group N and 162 patients in group E were analyzed. Flow diagram for treatment and characteristics of the patients are shown in Fig. 1 and Additional file 1: Table S2. ITT eradication rates were 84.4% (232/275) in group N and 74.7% (121/162) in group E, and there was a significant difference between the two groups (*P* = 0.02). According to the number of eradications, only the success rate for the 3rd-line eradication in group E was significantly lower than that in group N (59.7% vs 76.5%, *P* = 0.03). There was no significant difference in adverse events associated with eradication therapy between the two groups.

### Upper GI symptoms before and just after eradication therapy

Eighty-five participants did not fill out the questionnaire, and data for 343 patients including 302 patients in whom eradication was successful and 41 patients in whom eradication therapy failed were analyzed (Fig. 1).

In the patients in whom eradication therapy failed, there was no significant difference in GOS scores before and after eradication therapy: 1.80 ± 1.11 before and 1.82 ± 0.80 after in group E (n = 20) (*P* = 0.94) and 2.13 ± 1.15 before and 1.84 ± 0.94 after in group N (n = 21) (*P* = 0.06).

In the patients in whom *H. pylori* was eradicated, there were no significant differences between the two groups in total GOS score and score of each item before eradication. Successful eradication significantly improved upper GI symptom (Additional file 1: Table S3).

According to our definition of dyspepsia, 74 (36.5%) of the patients in group N and 31 (31.3%) of the patients in group E had dyspepsia before eradication. Within 2 months after successful eradication, 75.6% (56/74) of the patients in group N and 64.5% (20/31) of the patients in group E had improvement in dyspepsia (*P* = 0.34) (Table 1).

**Table 1:** Dyspepsia before and after successful eradication

	Group N (n = 203)	Group E (n = 99)	*P*
Mean age at *H. pylori* eradication, years ± SD	51.4 ± 9.9	70.6 ± 4.85	–
Sex (men/women)	87/116	34/65	0.17
Patients with dyspepsia before eradication, n (%)	74 (36.5)	31 (31.3)	0.44
Improvement in dyspepsia after eradication, n (%)	56/74 (75.6)	20/31 (64.5)	0.34

### Long-term effects of *H. pylori* eradication on dyspepsia

We sent the 3rd questionnaire to 105 patients who had been defined as having dyspepsia before eradication and in whom *H. pylori* had been eradicated. Responses to the questionnaire were obtained from 40 patients in group N and 20 patients in group E. Mean periods from successful eradication to filling out the questionnaire were 61.3 (15–124) months in group N and 54.8 (12–116) months in group E (*P* = 0.51) (Table 2).

**Table 2:** Long-term follow-up of dyspepsia symptoms after successful eradication

	Group N (n = 40)	Group E (n = 20)
Sex (men/women), n	17/23	9/11
Mean age at *H. pylori* eradication, years (range)	52.4 (17–64)	70.0 (65–76)
Mean period between eradication and 3rd questionnaires, months (range)	61.3 (15–124)	54.8 (12–116)
Patients who met the definition of *H. pylori-*associated dyspepsia, n (%)	32 (80)	12 (60)

**Fig. 2:**
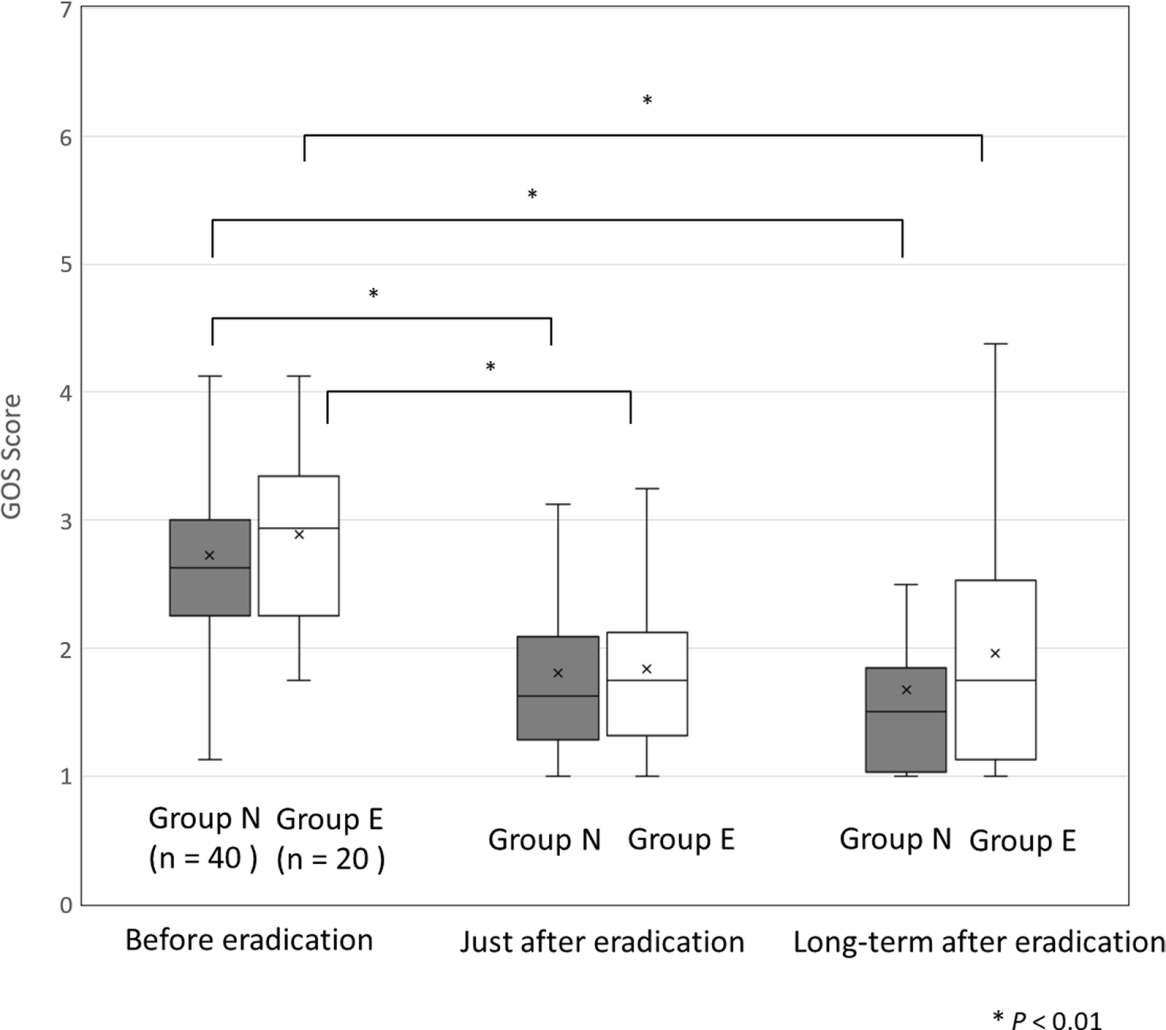
Changes in GOS scores based on questionnaires. There was no significant difference in GOS score between the two groups at any time point: 2.72 ± 0.93 in group N and 2.88 ± 0.67 in group E before eradication (*P* = 0.48), 1.83 ± 0.87 in group N and 1.84 ± 0.61 in group E just after eradication (at 1–2 months) (*P* = 0.88), 1.68 ± 0.88 in group N and 1.95 ± 1.00 in group E in the long term (*P* = 0.28). In the long term after successful eradication, GOS score was significantly decreased compared to that before eradication in both groups (*P* < 0.01)

**Fig. 3:**
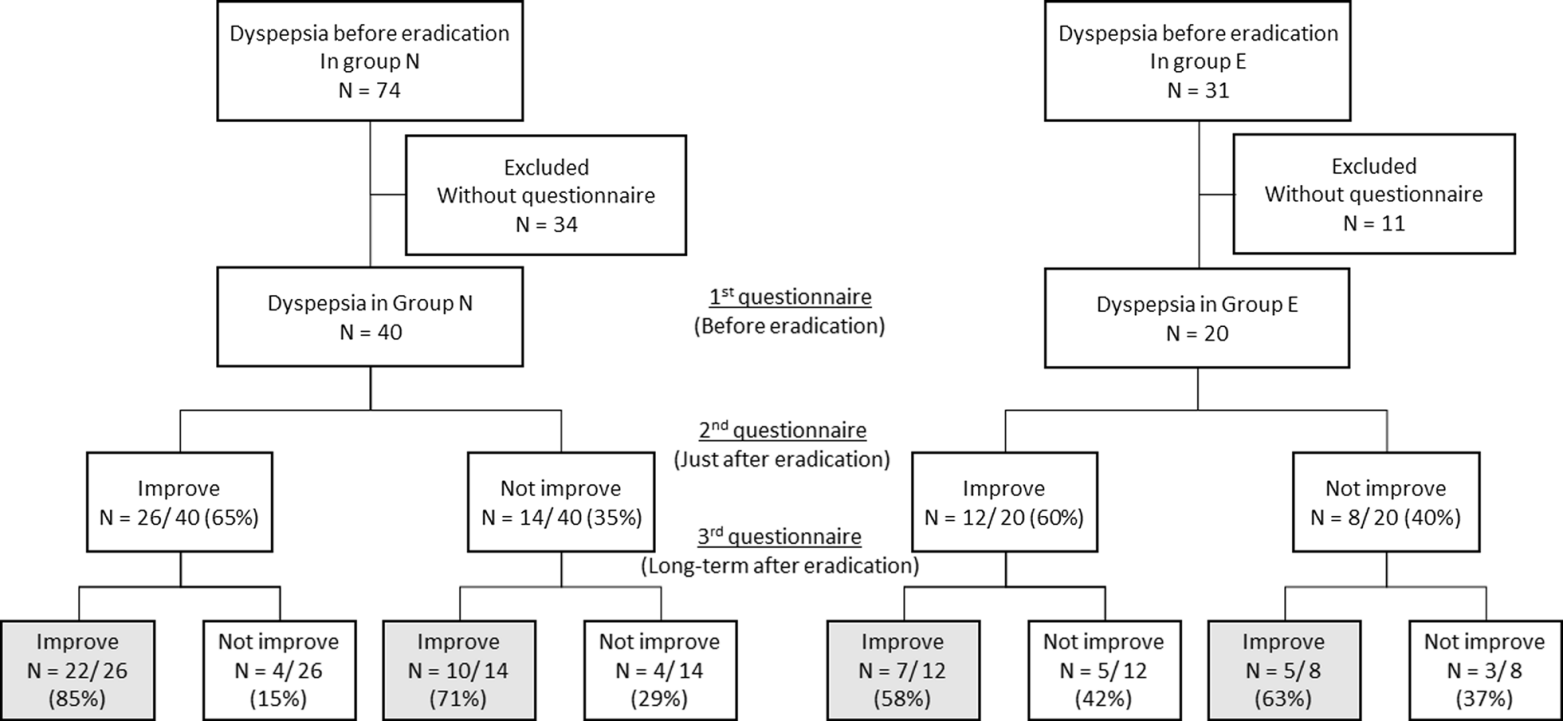
Long-term effects of *H. pylori* eradication on dyspepsia. In the non-elderly patients (group N), 32 patients, including 10 patients who did not show improvement just after eradication, had improvement in symptoms at the time of the 3rd questionnaire. In the elderly group (group E), 12 patients, including 5 patients who did not show improvement just after eradication, had improvement in symptoms at the time of the 3rd questionnaire. Relapse of symptoms occurred in 15% (group N) and 42% (group E) of the patients after judgment of eradication

GOS scores at the time of the 3rd questionnaire were significantly decreased compared to those before eradication in both groups (Fig. 2). Thirty-two patients (80%) in group N and 12 patients (60%) in group E had long-term improvement after eradication (*P* = 0.13) (Table 2). Short-term and long-term effects of eradication on dyspepsia symptoms were different in 35.0% of the patients in group N and 50.0% of the patients in group E (Fig. 3).

## Discussion

It has been shown that *H. pylori* eradication therapy is also effective for the elderly [7, 8, 15–17]. However, it has been reported that the prevalence of esophagitis and chronic gastritis and the function of the gastrointestinal tract are different in the elderly than in the non-elderly [10, 18]. There is no specific recommendation of *H. pylori* for elderly people in the Kyoto Global Consensus Report in 2015 [5]. The risks and benefits of eradication in the elderly, especially for dyspepsia, are unclear. Our study showed that eradication provided long-term relief of dyspepsia symptoms for elderly patients.

Dyspepsia symptoms often occur in *H. pylori*-positive individuals. Shimatani et al. reported that the proportion of patients with dyspeptic symptoms was significantly higher in *H. pylori*-positive patients than in *H. pylori*-negative patients (28.7% vs 6.5%) [19]. Kawamura et al. also reported that 46.3% of *H. pylori*-positive patients had dyspepsia symptoms [20]. Approximately 30% of our patients had dyspepsia symptoms, and the percentage is similar to that in previous studies.

It has been reported that *H. pylori* eradication therapy improved dyspepsia symptoms in 24–53% of patients [2, 21–24]. In our study, dyspepsia symptoms after successful eradication improved in about 73% of the patients in the long term, and the percentage of patients was slightly higher that in the previous studies (Fig. 3). Unfortunately, the definitions of improvement of dyspepsia were different in some studies, and further research is needed to compare the symptoms using the same methods at the same timing after eradication.

Tsuda et al. reported that a questionnaire within 2 months after *H. pylori* eradication might be useful for diagnosis in 70% of patients with HPD [25]. Similarly, questionnaires in the short term after eradication predicted HPD in 60% of elderly patients. However, the symptoms in 40% of the patients with dyspepsia changed in the long term and HPD could not be predicted. According to Kyoto Global Consensus Report, it is necessary to follow symptoms for more than 6 months after successful eradication to determine HPD as was indicated by our results [5]. The strength of our study was that the median time of the 3rd questionnaire was more than 4 years after eradication, and we were able to evaluate long-term effects of eradication on HPD.

*H. pylori* infection may cause dyspeptic symptoms through several mechanisms such as alterations of gastric acid secretion and persistent and active inflammation of the gastric mucosa [26]. Also, *H. pylori* may cause delays in gastric emptying and antral gastric secretion that are related to dyspeptic symptoms [27, 28]. There has been no report on a difference in the mechanisms between the elderly and non-elderly. Since *H. pylori* eradication often improves inflammation of the mucosa, acid secretion and gastric emptying often improve [29–31].

Our results showed that there was no significant difference in the improvement of dyspepsia between the elderly and non-elderly patients, though the elderly patients tended to have only slight improvement or relapse of dyspepsia symptoms. Atrophic gastritis, function of the gastrointestinal tract, medication, psychiatric illnesses, lifestyle with eating and exercise, and stress may be cause of such a difference [10, 18, 32, 33].

There have been a few studies on outcomes of eradication therapy for the elderly, but the outcomes investigated in those studies were for 1st-line and 2nd-line therapies [7, 8, 15, 34]. In our study, there was a significant difference in eradication rates only in the 3rd-line therapy. There were no significant differences in rates of eradication using PPIs and VPZ, and Kusunoki et al. and Nishida et al. reported that the effect of VPZ was unclear in elderly patients [15, 34]. Resistance to clarithromycin might be the main reason for failure of 3rd-line therapy, but that was unfortunately not checked in our subjects [35, 36]. Also, adverse events of eradication therapy are one of the concerns for the elderly. We have not experienced serious adverse events in eradication therapy, but there has been a report of death in an elderly patient [37]. Therefore, it is necessary to pay attention to drug interaction, hepatorenal function and co-morbidities in eradication, especially for the elderly.

The present study has several limitations. This study was a retrospective study with a small sample size conducted at a single institution. Also, antibiotic resistance was not tested. Another limitation is that the 3rd questionnaire survey was carried out as a cross-sectional survey. A study conducted for a period of 7 years after *H. pylori* eradication showed a fluctuation in dyspeptic symptoms after *H. pylori* eradication [32]. Therefore, our results may not reflect a lasting improvement effect. Also, this retrospective observational study did not satisfy the expected sample size due to our strict definition of dyspepsia and the poor response rate in the 3rd survey (57%). Therefore, our results are limited and further study with a large sample size is needed.

## Conclusion

Eradication of *H. pylori* age-independently improved dyspepsia symptoms. A test and treat strategy for *H. pylori* may be recommended for elderly patients with dyspepsia symptoms.

## Supplementary Information

**Additional file 1.** **Supplement table 1.** Regimens of *Helicobacter pylori* eradication. **Supplement table 2.** Characteristics of patients. **Supplement table 3.** Score of abdominal symptoms before and after successful eradication.

## Data Availability

The datasets used and/or analysed during the current study are available from the corresponding author on reasonable request.
