# Supplementary material for: Eradication of Helicobacter pylori may improve dyspepsia in the elderly for the long term
Source: BMC Gastroenterol. 2021 Nov 25;21:445. doi: 10.1186/s12876-021-02027-6 (PMC8620963; doi:10.1186/s12876-021-02027-6)
Supplement: Supplementary file 1 — Additional file 1. Supplement table 1. Regimens of Helicobacter pylori eradication. Supplement table 2. Characteristics of patients. Supplement table 3. Score of abdominal symptoms before and after successful eradication. [file 12876_2021_2027_MOESM1_ESM.docx]

Supplement 1. Regimens of *Helicobacter pylori* eradication

| **Eradication** | **Antibiotics** | **Antacids** |
| --- | --- | --- |
| 1st and 2nd lines | AMPC 750mg and CAM 200mg, b.i.d. 7days | RPZ 20mg  or LPZ 30mg  or VPZ 20mg, b.i.d. |
|  | AMPC 750mg and MNZ 250mg, b.i.d. 7days |  |
| 3rd line | AMPC 500mg q.i.d. and STFX 100mg, b.i.d. 7days  AMPC 500mg q.i.d. and STFX 100mg, b.i.d. 14days |  |
| 4th line | STFX 100mg and MINO 250mg b.i.d. 7 days  and AMPC 500mg q.i.d. 14 days |  |
| For penicillin allergy | CAM 200mg and MNZ 250mg b.i.d.  MNZ 250mg and STFX 100mg b.i.d. |  |

RPZ: rabeprazole, LPZ: lansoprazole, VPZ: vonoprazan, AMPC: amoxicillin, CAM: clarithromaycin, MNZ: metronidazole, STFX: sitafloxacin, MINO: minomycin, b.i.d: bis in die, q.i.d: quarte in die

Supplement 2. Characteristics of patients.

|  | Group N (n = 275) | Group E (n = 162) | *P* |
| --- | --- | --- | --- |
| Sex (men: women), n | 116: 159 | 61: 101 | 0.37 |
| Mean age at *H. pylori* eradication, years (range) | 51.7 (17-64) | 70.6 (65-87) | < 0.01 |
| Gastric disease, n (%) |  |  |  |
| Chronic gastritis | 164 (59.6) | 100 (61.7) | 0.69 |
| Peptic ulcer scar | 43 (15.6) | 13 (8.0) | 0.03 |
| Gastric cancer post treatment | 9 (3.3) | 13 (8.0) | 0.04 |
| Eradication therapy, n (%) |  |  |  |
| 1^st^-line | 160 (58.1) | 80 (49.4) | 0.09 |
| 2^nd^-line | 25 (9.1) | 13 (8.0) | 0.86 |
| 3^rd^-line | 85 (30.9) | 62 (38.3) | 0.12 |
| 4^th^-line | 5 (1.8) | 7 (4.3) | 0.14 |
| Penicillin allergy, n (%) | 69 (25.1) | 35 (21.6) | 0.42 |
| PPI-based regimen | 186 (67.6) | 104 (64.2) | 0.47 |
| PCAB-based regimen | 89 (32.4) | 58 (35.8) | 0.47 |

PPI: proton-pump inhibitors, PCAB: potassium competitive acid blocker

Supplement 3. Score of abdominal symptoms before and after successful eradication

|  | **Group N (n = 203)** | |  | **Group E (n = 99)** | |  |
| --- | --- | --- | --- | --- | --- | --- |
|  | Before | After | *P* | Before | After | *P* |
| GOS score | 1.84 ± 0.78 | 1.46 ± 0.51 | < 0.01 | 1.90 ± 0.84 | 1.52 ± 0.56 | < 0.01 |
| Epigastric pain | 2.20 ± 1.42 | 1.67 ± 0.97 | < 0.01 | 2.17 ± 1.44 | 1.52 ± 0.87 | < 0.01 |
| Heartburn | 2.03 ± 1.20 | 1.55 ± 0.91 | < 0.01 | 2.14 ± 1.29 | 1.70 ± 0.94 | < 0.01 |
| Acid regurgitation | 1.77 ± 1.11 | 1.46 ± 0.80 | < 0.01 | 1.86 ± 1.08 | 1.61 ± 0.96 | < 0.05 |
| Bloating | 2.29 ± 1.30 | 1.66 ± 0.85 | < 0.01 | 2.26 ± 1.31 | 1.80 ± 1.02 | < 0.01 |
| Nausea | 1.64 ± 1.21 | 1.22 ± 0.53 | < 0.01 | 1.67 ± 1.05 | 1.23 ± 0.51 | < 0.01 |
| Belching | 1.61 ± 0.95 | 1.39 ± 0.77 | < 0.01 | 1.59 ± 0.78 | 1.39 ± 0.82 | < 0.05 |
| Early satiety | 1.52 ± 0.97 | 1.29 ± 0.54 | < 0.01 | 1.56 ± 0.93 | 1.33 ± 0.62 | < 0.05 |
| Postprandial fullness | 1.67 ± 0.98 | 1.45 ± 0.78 | < 0.01 | 1.85 ± 1.24 | 1.52 ± 0.93 | < 0.05 |
